# A case report of hepatic abscesses with soft tissue infection caused by methicillin resistant *Staphylococcus aureus* in a young child

**DOI:** 10.1097/MD.0000000000009260

**Published:** 2017-12-15

**Authors:** Xia Wu, Ying-Zi Ye, Chuan-Qing Wang, Ai-Min Wang, Lei-Yan He, Hui Yu

**Affiliations:** a Department of Infectious Diseases; b Department of Nosocomial Infection Control; c Clinical Microbiology Laboratory, Children's Hospital of Fudan University, Shanghai, China.

**Keywords:** hepatic abscess, methicillin-resistant *Staphylococcus aureus*, soft tissue infection

## Abstract

**Rationale:**

Pyogenic hepatic abscess in children is a rare clinical condition. Hepatic abscesses caused by methicillin resistant *Staphylococcus aureus* are extremely rare.

**Patient concerns:**

A 6-year-old boy was referred to a tertiary children's hospital for a 6-day history of right lower abdominal pain and fever. Radiographic findings showed hepatic abscesses and soft tissue abscesses around the left femur.

**Diagnoses:**

Bacteriology of blood, hepatic abscesses, and soft tissue abscesses showed methicillin resistant *Staphylococcus aureus*.

**Interventions:**

Our patient received adequate drainage of MRSA abscesses and a complete course of antibiotics.

**Outcomes:**

The hepatic abscesses were healed and no recurrence has been founded until now.

**Lessons:**

This report describes an extremely rare case of hepatic abscesses with soft tissue infection caused by MRSA. Adequate drainage and appropriate systemic antibiotics should be considered as a standard treatment of MRSA abscesses in order to reduce the mortality rate and improve the quality of life.

## 1 Introduction

Methicillin resistant *Staphylococcus aureus* (MRSA) is an important pathogen, causing various infectious diseases from skin and soft tissue infections (SSTIs) to septicemia.^[1,2]^ Healthcare-associated MRSA (HA-MRSA) causes pneumonia and sepsis usually. In contrast, community-acquired MRSA (CA-MRSA) often cause SSTIs. Pyogenic hepatic abscess in children is a rare clinical condition. Less than 10% of hepatic abscesses are caused by *S aureus*, and fewer are caused by MRSA.^[3]^ We report a young boy who was successfully treated for hepatic abscesses with soft tissue infection caused by MRSA.

## 2 Case presentation

Our study was approved by Institutional Ethic Committee of Children's Hospital of Fudan University. Due to the retrospective nature of the study, informed consent was waived.

**Figure 1:**
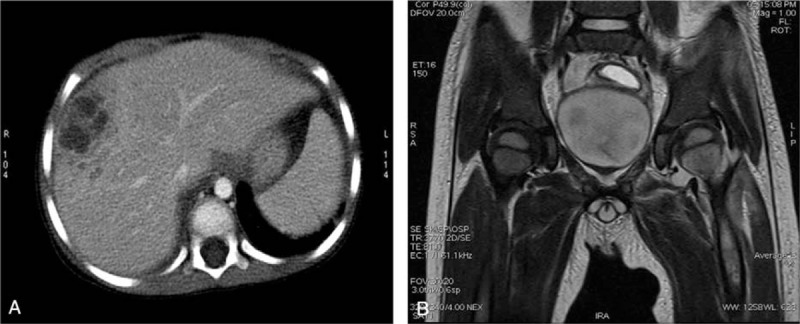
(A) Contrast-enhanced computed tomography showed multiple hepatic abscesses. (B) Magnetic resonance imaging revealed inflammation of soft tissue around left hip joints.

A 6-year-old boy was referred to a tertiary children's hospital for a 6-day history of right lower abdominal pain and fever. A physical examination showed that the abdomen was soft without palpable masses and the hepatic edge was palpable 4 cm below the costal margin and mildly tender. Blood tests revealed severe inflammation with a white blood cell (WBC) count of 16.8 × 10^9^/L, an elevated C-reactive protein level (143 mg/L), and an elevated erythrocyte sedimentation rate (105 mm/h). Other laboratory workup included liver enzymes, bilirubin, immune function, all of which were within normal limits. Contrast-enhanced computed tomography (CT) showed multiple heterogeneous lesions, suggesting hepatic abscesses (Fig. 1A). Magnetic resonance imaging (MRI) showed inflammation of soft tissue around the left hip joint (Fig. 1B). Blood culture was positive for MRSA and the final culture results showed sensitivity to gentamicin, levofloxacin, linezolid, rifampicin, fosfomycin, and vancomycin.

The patient was initially started on sulbactam sodium and cefoperazone sodium for 1 week (50 mg/kg, q8h), but switched to intravenous vancomycin (15 mg/kg, q8h) after susceptibilities of the blood culture isolate were known. However, the patient's fever and radiographic findings did not improve after 10 days of treatment. The patient then underwent percutaneous transhepatic abscess drainage. However, the patient's clinical condition deteriorated with continued high fevers (39°C) and worsening pain in the left thigh. Urgent incision and drainage of soft tissue abscesses around the left femur were performed. Bacteriology of hepatic abscesses and soft tissue abscesses showed MRSA, of which sensitivity to antibiotics was similar to that of blood culture. The patient became afebrile after 4 days of drainage of soft tissue abscesses around the left femur.

**Figure 2:**
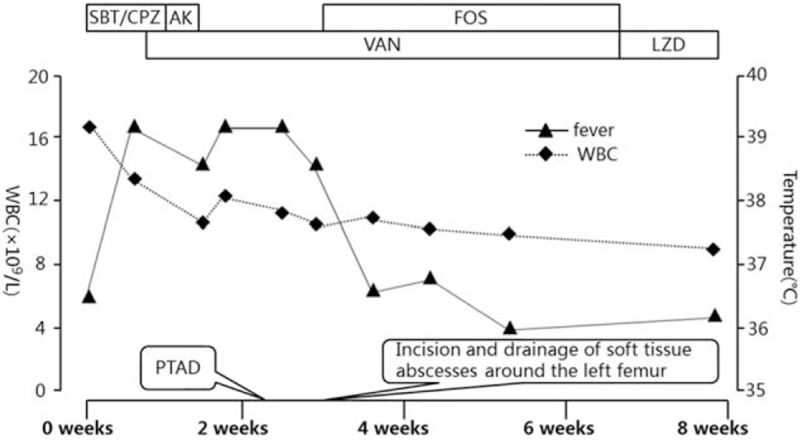
Summarization of the treatment course in the patient. AK = amikacin, FOS = fosfomycin, LZD = linezolid, PTAD = percutaneous transhepatic abscess drainage, SBT/CPZ = sulbactam sodium and cefoperazone sodium, VAN = vancomycin.

The treatment course of the patient is shown in Fig. 2. After discharge, the patient was followed in the outpatient clinic with clinical, blood tests, radiological controls. The patient was in good condition and free of pain. Follow-up ultrasonography performed 7 weeks after the initial percutaneous drainage showed resolution of hepatic abscesses. During 2 years of follow-up, the patient remains in good health, with mild slippage of the left femoral head.

## 3 Discussion

The typical presentation of hepatic abscesses in children is right upper quadrant pain and fever and laboratory investigations show leukocytosis. Ultrasonography, CT, and MRI play important roles in the diagnosis if hepatic abscess is suspected.^[4]^ Patients can also undergo CT and ultrasound guided drainage of the abscess. Etiologies of the hepatic abscess have considerably changed over the past several decades.^[5]^ The most common bacteria associated with pyogenic hepatic abscess include *Escherichia coli*, group D *Streptococcus*, and *Klebsiella pneumoniae*. Hepatic abscess due to MRSA is uncommon and recovery of hepatic abscess from MRSA is extremely rare. The first priority when blood culture is positive for MRSA in a febrile child is to investigate the source of MRSA.

Treatment of hepatic abscess involves drainage and antibiotic therapy.^[6–8]^ After empiric antibiotics treatment has been initiated, we must follow the culture results and start a targeted antibiotic therapy. In the present case, although sensitivity of MRSA to vancomycin was clear, hepatic abscesses did not disappear with antibiotic treatment alone. Some reports have emphasized the effectiveness of antibiotic therapy alone in the treatment of hepatic abscess,^[9]^ but this therapy should be reserved for those in whom the abscess is small. Therefore, in our patient, additional interventional treatment with percutaneous transhepatic abscess drainage was performed, which failed to relieve the high fever. The most likely explanation as to why our patient failed to respond to initial therapy is formation of soft tissue abscesses around the left femur. The satisfactory clinical result after incision and drainage of soft tissue supports this possibility.

This case also highlights the problem of CA-MRSA. Most CA-MRSA possess various virulence, such as Panton–Valentine leukocidin (PVL).^[10]^ PVL-positive CA-MRSA contributes to severe SSTIs. CA-MRSA is more likely to cause cellulitis and abscesses than HA-MRSA, and is more susceptible to multiple antibiotics, such as ciprofloxacin and clindamycin.^[11]^ In our patient, the aggressive nature of abscesses suggests that the most possible cause of MRSA abscess in this patient was from hematogenous spread, which come from soft tissue infection. The MRSA isolate is susceptible to gentamicin, levofloxacin, linezolid, rifampicin, fosfomycin. The course of antibiotic therapy for hepatic abscesses is generally 2 to 6 weeks.^[12]^ Our patient recovered after adequate drainage of MRSA abscesses and a complete course of antibiotics. There was no recurrence in this case in the last follow-up at 2 years.

## 4 Conclusion

In conclusion, we present a rare case of hepatic abscesses with soft tissue infection caused by MRSA. MRSA must be considered as a possible source of hepatic abscess in patients with SSTIs, especially without obvious gastrointestinal sources. Adequate drainage and appropriate systemic antibiotics should be considered as a standard treatment of MRSA abscesses in order to reduce the mortality rate and improve the quality of life.
